# Contributions of the US Centers for Disease Control and Prevention in Implementing the Global Health Security Agenda in 17 Partner Countries

**DOI:** 10.3201/eid2313.170898

**Published:** 2017-12

**Authors:** Arthur G. Fitzmaurice, Michael Mahar, Leah F. Moriarty, Maureen Bartee, Mitsuaki Hirai, Wenshu Li, A. Russell Gerber, Jordan W. Tappero, Rebecca Bunnell, GHSA Implementation Group

**Affiliations:** Author affiliation: Centers for Disease Control and Prevention, Atlanta, Georgia, USA

**Keywords:** global health, global health security, Global Health Security Agenda, public health, emergency response, emergency management, CDC, GHSA, International Health Regulations, IHR 2005, United States

## Abstract

The Global Health Security Agenda (GHSA), a partnership of nations, international organizations, and civil society, was launched in 2014 with a mission to build countries’ capacities to respond to infectious disease threats and to foster global compliance with the International Health Regulations (IHR 2005). The US Centers for Disease Control and Prevention (CDC) assists partner nations to improve IHR 2005 capacities and achieve GHSA targets. To assess progress through these CDC-supported efforts, we analyzed country activity reports dating from April 2015 through March 2017. Our analysis shows that CDC helped 17 Phase I countries achieve 675 major GHSA accomplishments, particularly in the cross-cutting areas of public health surveillance, laboratory systems, workforce development, and emergency response management. CDC’s engagement has been critical to these accomplishments, but sustained support is needed until countries attain IHR 2005 capacities, thereby fostering national and regional health protection and ensuring a world safer and more secure from global health threats.

Recent infectious disease outbreaks have demonstrated that a local threat can rapidly become a global crisis that jeopardizes the health, economy, and safety of persons everywhere. Severe outbreaks and regional epidemics, including severe acute respiratory syndrome, Middle East respiratory syndrome, Ebola virus disease (EVD), Zika virus, and novel influenza viruses, have highlighted the importance of countries developing core capacities to contain public health threats, as outlined in the International Health Regulations (IHR 2005) (*1*–*3*). As of 2014, fewer than a third of 196 countries reported achieving IHR 2005 capacities (*4*). The Global Health Security Agenda (GHSA), a partnership of nations, international organizations, and civil society, was launched in 2014 with the mission to build countries’ capacities to respond to infectious disease threats, thereby progressing toward IHR 2005 compliance (*5*). Global health security relies on all countries building IHR 2005 capacities to rapidly detect and control public health threats at their sources.

GHSA is built on 3 pillars: 1) prevent avoidable epidemics; 2) detect threats early; and 3) respond rapidly and effectively. To date, 61 countries have joined GHSA, including approximately a dozen countries partnering with low- and middle-income countries to assist in their GHSA work. In 2014, the United States committed to working with 31 partner countries and the Caribbean community to meet targets associated with each of 11 technical areas (termed Action Packages) that align with GHSA’s 3 pillars (*6*). Through GHSA, the United States has committed technical and fiscal support to a subset of 17 countries termed Phase I and technical assistance with work plan development in Phase II countries. Exceeding this commitment, the US Centers for Disease Control and Prevention (CDC) works to strengthen global health security capacities in approximately 3 dozen countries, including Phase I and Phase II countries, as well as Ebola preparedness countries, which surround those countries affected by the recent EVD outbreak (Figure). CDC works across all 11 GHSA technical areas, with a special emphasis on 4 that serve as a platform for public health emergencies and health security: surveillance, laboratory systems, workforce development, and emergency response management. CDC staff stationed in partner countries, with support from CDC headquarters–based subject matter experts and funded partners, provide direct technical assistance to partnering government counterparts (*7*–*9*). CDC’s goal is to help countries achieve GHSA and IHR 2005 targets by strengthening sustainable systems and capacities to respond to health threats locally, thereby preventing the spread of disease and protecting persons in the United States and around the world from outbreaks and other public health threats. Descriptions of CDC’s early GHSA work with counterparts in Uganda and Vietnam have been published (*10*,*11*), but substantial progress has been made across all Phase I countries. Here we document the major GHSA accomplishments that these 17 countries achieved with CDC support during April 2015–March 2017. These successes are now informing ongoing program implementation in these and other countries.

**Figure:**
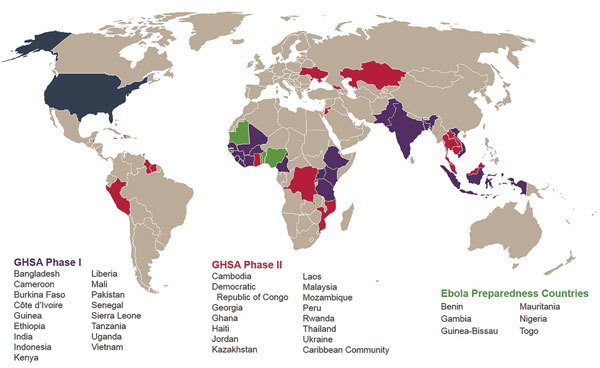
GHSA countries supported by the US Centers for Disease Control and Prevention. GHSA, Global Health Security Agenda.

## Methods

In January 2015, CDC technical staff commenced working with ministries of health (MOHs) and other partner country counterparts to assess baseline capacities related to 11 GHSA technical areas. By June 2015, annual country work plans had been developed, detailing activities through which CDC would assist countries in achieving their first-year objectives in each technical area. The level and nature of CDC support varied across activities depending on technical assistance needs, inputs from other collaborators, and host country and donor financing. CDC staff reported on activity progress on a quarterly basis. Reports indicated the status (i.e., completed, on track, delayed, or canceled) and described progress toward completion of each work plan activity. Reporting information was provided to CDC headquarters–based evaluators 4 times: December 2015, April–May 2016, July–August 2016, and October–November 2016. Results were used to improve and update work plans.

Trained CDC evaluators analyzed quarterly reporting data by technical area, objectives within technical areas, and activities within objectives. In May 2016, CDC evaluators analyzed reporting information for completed activities across all 17 Phase I countries and grouped results into the following categories: 1) real-time surveillance and reporting; 2) national laboratory system and biosafety/biosecurity; 3) workforce development; and 4) emergency management and Emergency Operations Centers (EOCs). This organizational framework reduced the likelihood of missing information because of misclassification, such as if different countries reported related activities in similar, but different, technical areas. For example, national laboratory system and biosafety/biosecurity activities were batched for analyses to ensure all relevant laboratory activities were analyzed together. Activities in other technical areas were analyzed in November 2016.

A CDC evaluator analyzed the completed activity descriptions, objective descriptions, and activity progress data across all Phase I countries for each of the 4 categories. A second CDC evaluator reviewed and validated the first evaluator’s analyses; discrepancies were discussed and resolved by a CDC subject matter expert familiar with GHSA technical areas and overseeing all analyses for consistency. Completed activities were summarized by using common terminology for similar major accomplishments achieved by countries with CDC support. (Data on accomplishments achieved by <6 countries are available but not shown here.) Evaluators provided CDC headquarters and field staff with lists of countries that had achieved each major accomplishment, so they could add a country that had not been identified through reporting data analyses or remove a country from an accomplishment category if appropriate. CDC field staff worked with MOHs in some countries to confirm that the revised language accurately reflected country progress.

In November 2016, this process was repeated for completed activities in all 11 technical areas, resulting in a final list that integrated all accomplishments organized into 4 categories (Tables 1–4). In April 2017, CDC field staff in all 17 countries confirmed that the partner country had achieved these major accomplishments with CDC assistance during April 2015–March 2017. CDC evaluators determined the number and proportion of countries that achieved each accomplishment with CDC support.

**Table 1:** Key CDC-supported accomplishments toward achieving GHSA targets related to real-time surveillance in 17 Phase I countries, 2015–2017*

GHSA targets and CDC-supported accomplishments	Related JEE indicators (*12*)	No. countries
**Strengthened foundational indicator-and event-based surveillance systems that are able to detect events of significance for public health, animal health, and health security**		
Surveillance systems		
Established systems and mechanisms at national or subnational levels for detecting public health events from a variety of sources	D.2.1	16
Improved timeliness or geographic coverage of routine public health threat reporting	D.2.2, D.3.2	11
Expanded surveillance systems for >3 syndromes indicative of potential public health emergencies (e.g., severe acute respiratory syndrome, acute flaccid paralysis, acute hemorrhagic fever, acute watery diarrhea with dehydration, jaundice with fever)	D.2.4	13
Expanded surveillance systems for zoonotic diseases to include additional pathogens or broader geographic coverage	P.4.1, D.2.1	13
Expanded surveillance systems to include additional pathogens that cause vaccine-preventable diseases	D.2.1	10
Conducted community immunizations in response to vaccine-preventable disease surveillance information	P.7.1, D.2.3	13
Expanded surveillance systems for antimicrobial resistance to include additional pathogens or broader geographic coverage	P.3.2, D.2.1	7
Strategic planning and assessment		
Developed plans to improve the flow and timing of surveillance information and reporting	D.2.2, D.2.3, D.3.1, D.3.2	10
Assessed immunization surveillance, case management, and reporting systems	D.2.2	11
Assessed antimicrobial resistance and drug-resistant tuberculosis surveillance and reporting capacity	P.3.2, D.3.2	9
Training		
Participated in >1 levels of FETPs	D.4.1	17
Integrated FETP trainees into core public health surveillance functions	D.2.3	15
**Improved communication and collaboration across sectors and between subnational, national, and international levels of authority regarding surveillance of events of public health significance**		
Strategic planning and assessment		
Identified national policies, legal authorities, and gaps for the conduct of public health surveillance	P.1.1, P.1.2, D.2.1, D.2.2, D.2.4	17
Identified subnational units responsible for indicator- and event-based surveillance	D.2.1	13
Documented national priority public health threats or completed risk assessment	D.2.3, R.1.2	9
Multisectoral coordination		
Developed plans to implement a joint system for surveillance with defined roles, responsibilities, operational processes, and procedures for priority diseases with ministries of health, agriculture, and defense	D.2.1, D.2.2, P.2.1, P.4.1	7
Developed plans and procedures for surveillance capacity for port health services at points of entry	R.3.1, D.2.1, PoE.1	8
Training		
Trained community members to detect and report potential health threats	D.2.1, D.3.2	14
**Improved country and regional capacity to analyze and link data from and between strengthened, real-time surveillance systems, including interoperable, interconnected electronic reporting systems**		
Strategic planning and assessment		
Assessed reporting systems for development of the national surveillance plan	D.2.2, D.3.1, D.3.2	10
Documented gaps in surveillance data collection, analysis, and interpretation capabilities	D.2.3	12
Developed plan for interoperable information systems supporting indicator- or event-based surveillance and data exchange and integration for priority diseases	D.2.1, D.2.2	8
Training		
Developed training curriculum for health systems personnel in surveillance methods and data use	D.2.1, D.2.2, D.2.3, D.2.4	16
Trained surveillance staff to ensure best practices according to International Health Regulations standards	D.2.1, D.2.2, D.2.3, D.2.4, D.4.1	9

*Countries: Bangladesh, Burkina Faso, Cameroon, Côte d’Ivoire, Ethiopia, Guinea, India, Indonesia, Kenya, Liberia, Mali, Pakistan, Senegal, Sierra Leone, Tanzania, Uganda, and Vietnam. CDC, US Centers for Disease Control and Prevention; FETP, Field Epidemiology Training Program; GHSA, Global Health Security Agenda; JEE, Joint External Evaluation tool.

**Table 4:** Key CDC-supported accomplishments toward achieving GHSA targets related to emergency management in 17 Phase I countries, 2015–2017*

GHSA target and CDC-supported accomplishments	Related JEE indicators (*12*)	No. countries
**Public health EOC functioning according to minimum common standards**		
Strategic planning and assessment		
Identified national policies, legal authorities, and gaps for the conduct of public health emergency response	P.1.1, P.1.2, R.1.1, R.1.2, R.2.1, R.2.2, R.2.4	17
Assessed baseline of national public health emergency management capacities	R.1.2, R.2.1	14
Documented national priority public health threats or completed risk assessment	D.2.3, R.1.2	9
EOC facility		
Obtained buy-in from country leadership for permanent EOC facility and associated program	R.2.1, R.2.2	15
Identified facility location or funding mechanisms for EOC	R.2.2	16
Developed EOC policies, plans, protocols, or standard operating procedures	R.2.2, R.2.4	15
Multisectoral coordination		
Operationalized multisectoral One Health mechanisms to limit animal-to-human spillover of zoonotic diseases	P.4.3, P.2.1	9
Initiated activities to strengthen response coordination (e.g., through MOUs) across public health, animal health, law enforcement, and other sectors	R.3.1, R.1.1, P.2.1, P.4.3, PoE.2	12
Identified points of contact and informal process for communication and information-sharing across public health, animal health, law enforcement, and other sectors	R.3.1, P.4.3, P.2.1, PoE.2	13
Improved logistics planning to deploy staff, medicines, and supplies during a public health emergency	R.4.1, R.4.2, R.1.1, PoE.1	10
**Trained EOC staff capable of activating a coordinated emergency response within 120 minutes of the identification of a public health emergency**		
Strategic planning and assessment		
Identified needs for core public health emergency management staff	R.2.1, D.4.1, R.1.1	15
Assessed baseline capacity of partnering agencies for response to a biologic threat	P.2.1, R.3.1	12
Training		
Activated EOC for an exercise or real emergency response	R.2.3, R.3.1	11
Trained EOC staff in public health emergency management (basic level)	R.2.1, D.4.1	14
Committed to train EOC staff through CDC’s Public Health Emergency Management Fellowship	R.2.1, D.4.1	16
Recruited key staff for public health emergency management	R.2.1, D.4.1	13

*Countries: Bangladesh, Burkina Faso, Cameroon, Côte d’Ivoire, Ethiopia, Guinea, India, Indonesia, Kenya, Liberia, Mali, Pakistan, Senegal, Sierra Leone, Tanzania, Uganda, and Vietnam. CDC, US Centers for Disease Control and Prevention; EOC, Emergency Operations Center; GHSA, Global Health Security Agenda; JEE, Joint External Evaluation tool; MOU, memo of understanding.

## Results

Overall, our analysis found that CDC supported 675 accomplishments across all 11 GHSA technical areas in 17 Phase I countries. These accomplishments reflect achievements in >6 countries (Tables 1–4). Eleven countries each achieved >40 of these accomplishments, and each of the 17 countries achieved >18.

### Disease and Syndromic Surveillance

#### Surveillance Systems

With CDC’s technical assistance, 16 countries established real-time surveillance systems and mechanisms for detecting potential public health events at the national or subnational level. Surveillance systems were improved for zoonotic diseases (13 countries), vaccine-preventable diseases (10 countries), and antimicrobial resistance (7 countries). Thirteen countries met GHSA targets for real-time surveillance of >3 syndromes indicative of potential public health emergencies (e.g., severe acute respiratory syndrome, acute flaccid paralysis, acute hemorrhagic fever, acute watery diarrhea with dehydration, and jaundice with fever). In 11 countries, CDC helped countries expand and enhance previously established indicator-based surveillance systems to capture potential threats from larger geographic areas and improve timeliness. CDC supported community immunizations in response to surveillance data on vaccine-preventable diseases in 13 countries (Table 1).

#### Surveillance Strategic Planning

CDC identified national policies, legal authorities, and gaps for conducting public health surveillance in each of the 17 Phase I countries. In 13 countries, CDC worked with MOHs to determine the appropriate level of subnational jurisdictions (e.g., districts) for reporting surveillance information to the national MOH. Plans and procedures for multisectoral surveillance were developed with ministries of health, agriculture, and defense in 7 countries and with port health services for national points of entry in 8 countries (Table 1).

CDC assisted 12 countries in documenting gaps in surveillance data collection, analysis, and interpretation capabilities; 8 of these countries developed plans for improving interoperability of disparate surveillance systems to better integrate available data from different sources. Eleven countries conducted specialized assessments for immunization surveillance and 9 for antimicrobial resistance (e.g., drug-resistant *Mycobacterium tuberculosis*) surveillance (Table 1).

### National Laboratory System

#### Laboratory Confirmation of Outbreaks

CDC trained laboratory technicians in all 17 Phase I countries and provided 16 countries with new laboratory diagnostics to confirm potential outbreaks identified by surveillance systems, focusing on priority pathogens (e.g., influenza virus, poliovirus, HIV, *M. tuberculosis, Salmonella enterica* serovar Typhi, *Plasmodium* sp., and *Vibrio cholerae*). CDC worked with 9 countries to assess diagnostic capabilities for priority pathogens and 10 countries for antimicrobial resistance. CDC assisted 9 countries in establishing new systems for transporting specimen samples to national reference laboratories (Table 2).

**Table 2:** Key CDC-supported accomplishments toward achieving GHSA targets related to national laboratory systems in 17 Phase I countries, 2015–2017*

GHSA target and CDC-supported accomplishments	Related JEE indicators (*12*)	No. countries
**Real-time biosurveillance with a national laboratory system**		
Strategic planning and assessment		
Identified national policies, legal authorities, and gaps for the conduct of a national public health laboratory system	P.1.1, P.1.2, D.1.2, D.1.3, D.1.4	17
Operationalized national plan of action with internationally accepted best practices for priority diseases	D.1.1, D.1.2, D.1.3, D.1.4	11
Developed tier-specific testing strategies for priority diseases at designated laboratories	D.1.3	10
Specimen referral system		
Established functional system for specimen transport to reference laboratories within the appropriate timeframe of collection	D.1.2	9
Conducted investigations or training exercises to confirm functionality of specimen referral systems	D.1.2	8
Training		
Trained laboratory technicians	D.1.1, D.1.3	17
**Effective modern point-of-care and laboratory-based diagnostics**		
Strategic planning and assessment		
Assessed diagnostics, data quality, and staff performance	D.1.1, D.1.3, D.1.4	9
Assessed antimicrobial resistance and drug-resistant tuberculosis laboratory capacity	P.3.1	10
Diagnostics		
Acquired new diagnostic equipment and capabilities (e.g., specimen test kits) to detect priority pathogens (e.g., influenza virus, poliovirus, HIV, *Mycobacterium tuberculosis*, *Salmonella enterica* serovar Typhi, *Plasmodium* sp., *Vibrio cholerae*)	D.1.1, D.1.3	16
**Whole-of-government national biosafety and biosecurity system is in place, ensuring that especially dangerous pathogens are identified, held, secured, and monitored in a minimal number of facilities according to best practices; biologic risk management training and educational outreach are conducted to promote a shared culture of responsibility, reduce dual-use risks, mitigate biologic proliferation and deliberate use threats, and ensure safe transfer of biologic agents; and country-specific biosafety and biosecurity legislation, laboratory licensing, and pathogen control measures are in place as appropriate**		
Biosafety and biosecurity		
Trained staff on biosafety and biosecurity	P.6.2	15
Identified staff in ministries of health, agriculture, and defense responsible for inspection or certification of laboratories for compliance with biosafety and biosecurity requirements	P.6.1	8
Inventoried dangerous pathogens and developed a plan to manage them	P.6.1	6

*Countries: Bangladesh, Burkina Faso, Cameroon, Côte d’Ivoire, Ethiopia, Guinea, India, Indonesia, Kenya, Liberia, Mali, Pakistan, Senegal, Sierra Leone, Tanzania, Uganda, and Vietnam. CDC, US Centers for Disease Control and Prevention; GHSA, Global Health Security Agenda; JEE, Joint External Evaluation tool.

#### Biosafety and Biosecurity

CDC provided technical assistance to 6 countries to inventory dangerous pathogens and develop plans to manage them in their national laboratory systems. CDC helped 15 countries train technical and administrative staff on biosafety and biosecurity. Eight countries identified staff in the ministries of health, agriculture, and defense responsible for inspecting and certifying laboratories for biosafety and biosecurity compliance (Table 2).

### Workforce Development

#### Field Epidemiology Training Programs

All 17 Phase I countries now participate in basic-level frontline (3-month training), intermediate (6- to 9-month training), or advanced (2-year training) Field Epidemiology Training Programs (FETPs) (*13*–*15*) (Table 3). These field-based, CDC-supported programs train members of a nation’s health workforce to become disease detectives at national and subnational levels. Since April 2015, CDC has established 14 new frontline and 2 new FETP-Advanced in Phase I countries. Trainees from all countries investigated real or potential outbreaks as part of their training. Numbers of trainees per country ranged from 24 to 622; nearly half of trainees were frontline surveillance officers (*16*).

**Table 3:** Key CDC-supported accomplishments toward achieving GHSA targets related to workforce development in 17 Phase I countries, 2015–2017*

GHSA target and CDC-supported accomplishments	Related JEE indicators (*12*)	No. countries
**Workforce including physicians, veterinarians, biostatisticians, laboratory scientists, farming and livestock professionals, and field epidemiologists who can systematically cooperate to meet relevant International Health Regulations and performance of veterinary services core competencies**		
Strategic planning and assessment		
Created national, multisectoral workforce development strategic plan	D.4.3	6
Assessed country's public health training programs, education system, and workforce gaps	D.4.1, D.4.3	15
Assessed country's current status of One Health workforce	P.4.2, D.4.1	8
Identified needs for core public health emergency management staff	R.2.1, D.4.1, R.1.1	15
Assessed laboratory staff performance	D.1.4	9
Identified staff in ministries of health, agriculture, and defense responsible for inspection or certification of laboratories for compliance with biosafety and biosecurity requirements	P.6.1	8
FETP		
Conducted 3-month FETP-Frontline	D.4.2	15
Conducted intermediate or FETP-Advanced (6 months–2 years)	D.4.2	11
Participated in FETP-Intermediate or FETP-Advanced run by another country	D.4.2	6
Provided FETP to >1 staff member from >50% of subnational jurisdictions	D.4.1, D.4.2	6
Integrated FETP trainees into core public health functions	D.4.1, D.2.3	15
Other training		
Conducted public health multidisciplinary (e.g., One Health) trainings	P.4.2	13
Trained laboratory technicians	D.1.1, D.1.3	17
Trained staff on biosafety and biosecurity	P.6.2	15
Developed infection prevention and control training programs, including antimicrobial resistance prevention	P.3.3	7
Trained community members to detect and report potential health threats	D.2.1, D.3.2	14
Developed training curriculum for health systems personnel in surveillance methods and data use	D.2.1, D.2.2, D.2.3, D.2.4	16
Trained surveillance staff to ensure best practices according to International Health Regulations standards	D.4.1, D.2.1, D.2.2, D.2.3, D.2.4	9
Activated EOC for an exercise or real emergency response	R.2.3, R.3.1	11
Trained EOC staff in public health emergency management (basic level)	R.2.1, D.4.1	14
Committed to train EOC staff through CDC’s Public Health Emergency Management Fellowship	R.2.1, D.4.1	16
Recruited key staff for public health emergency management	R.2.1, D.4.1	13

*Countries: Bangladesh, Burkina Faso, Cameroon, Côte d’Ivoire, Ethiopia, Guinea, India, Indonesia, Kenya, Liberia, Mali, Pakistan, Senegal, Sierra Leone, Tanzania, Uganda, and Vietnam. CDC, US Centers for Disease Control and Prevention; EOC, Emergency Operations Center; FETP, Field Epidemiology Training Program; GHSA, Global Health Security Agenda; JEE, Joint External Evaluation tool.

#### Additional Training

Other CDC-supported training activities addressed additional GHSA targets. In 14 countries, CDC worked with MOHs to train community leaders in event-based surveillance. In 16 countries, CDC helped develop training curricula for surveillance and data analysis methods in English or the predominant national language (i.e., French or Vietnamese). In 7 countries, CDC provided trainings and developed infection prevention and control programs for healthcare facilities to combat antimicrobial resistance. In 13 countries, CDC led multidisciplinary and multisectoral public health trainings, including One Health trainings for preventing zoonotic disease spillover from animals to humans (Table 3).

#### Workforce Strategic Planning

CDC supported 16 countries in strategic planning related to the national public and animal health workforce. CDC assisted 6 of these countries in creating national multisectoral workforce development strategic plans based on assessments of existing public health training programs, educational systems, and gaps in the national public health workforce (Table 3).

### Emergency Management and Response

#### EOCs

CDC worked with all 17 Phase I countries to improve public health emergency management capacities, such as by establishing EOCs and training EOC staff in incident management in 15 countries. Twenty-nine staff from 14 countries’ MOHs, national public health institutes, and other national and international organizations completed CDC’s Public Health Emergency Management Fellowship Program (*17*). CDC helped 15 countries develop EOC policies and protocols, and 11 countries activated the EOC for an exercise or real public health emergency response (Table 4).

#### Multisectoral Coordination

CDC provided assistance to 14 countries to complete public health risk assessments and document national priority public health threats. Nine countries established One Health mechanisms for joint response across human, animal, and environmental health sectors to prevent or limit animal-to-human spillover of zoonotic diseases (*18*). CDC worked with 13 countries to assess baseline capacities of agencies to respond to biologic threats across public health, animal health, law enforcement, and other sectors. CDC initiated activities to strengthen response coordination across multiple sectors in 12 countries and identified points of contact for multisectoral information-sharing in 10 countries (Table 4).

## Discussion

During April 2015–March 2017, CDC supported 17 Phase I countries in achieving 675 accomplishments in 11 GHSA technical areas. Although GHSA is still in early stages of implementation, CDC’s support to countries has helped improve their capabilities, especially in the cross-cutting areas of public health surveillance, national laboratory systems, workforce development, and emergency response management. Accomplishments in these technical areas have also contributed to the countries’ progress in the other GHSA technical areas and IHR 2005 core capacities.

Robust surveillance networks linked with laboratory testing can enable early detection of public health threats before they escalate into outbreaks and threaten communities, nations, and the world. CDC’s efforts to build country capacity to detect potential outbreaks focused on increasing the numbers of diseases captured by surveillance and reporting systems, expanding these systems to include additional subnational jurisdictions and community-level surveillance, and strengthening processes to improve the timeliness and efficiency of communication across all levels.

CDC worked with health, agriculture, defense, and other ministries to broaden the types of pathogens and syndromes that can be detected by improved surveillance systems. As a result of CDC’s GHSA work, countries that previously had systems to monitor a limited range of potential public health threats are now better able to detect animal-to-human disease spillover, healthcare-associated infections, and other potential outbreaks by monitoring more diseases and syndromes systematically and frequently. Early detection of public health threats can lead to timely interventions to prevent escalation into major outbreaks (*19*–*21*). Phase I countries have already used improved surveillance data to inform prevention efforts. For example, increased surveillance of vaccine-preventable diseases resulted in community immunizations to prevent further spread of measles and other diseases in 13 countries, including Guinea, Indonesia, and Liberia, where vaccination coverages are low. Furthermore, CDC worked with Phase I countries to incorporate hands-on experience investigating potential outbreaks into FETPs.

Surveillance capacity-building efforts also focused on expanding geographic coverage. Public health surveillance and laboratory capacity have typically been concentrated in urban centers, limiting countries’ abilities to detect outbreaks in rural areas (*20*,*22*). CDC assisted Phase I countries with establishing integrated surveillance systems that share data across healthcare facilities, subnational jurisdictions (e.g., districts), and MOHs. CDC helped countries train surveillance officers throughout multiple levels of countries’ health systems. In addition to training field epidemiologists through FETPs, CDC helped countries enlist the help of community leaders in detecting threats early by training them on community-based disease surveillance and reporting to complement healthcare facility surveillance. Community-level disease monitoring has been shown to influence intervention efforts and reduce the incidence of disease and prevalence of premature death. For example, community health workers in West Africa used surveillance data to target immunizations and reduce the number of cases of vaccine-preventable meningococcal disease by half (*23*,*24*). These efforts aim to prevent outbreaks at the source before spreading rapidly within large cities or to other countries.

National laboratory systems are integral for assessing public health threats and targeting outbreak response efforts. Laboratory testing of specimen samples is necessary to confirm suspected public health threats identified through disease and syndromic surveillance (*25*). Timely confirmation of public health threats relies upon laboratory systems that link central reference laboratories with peripheral laboratories, securely and rapidly transport specimens from patients to laboratories, and efficiently report accurate test results from laboratories to patients and MOHs (*26*). CDC’s assistance has been vital to providing countries with diagnostic capabilities and establishing specimen transport systems to decrease the time from specimen collection to testing at a certified national public health laboratory. This work is necessary to confirm public health threats so response efforts can be directed appropriately. CDC’s training of laboratory technicians will empower countries to confirm potential outbreaks of a broader set of pathogens more accurately and expediently.

CDC worked with other US government entities and partner countries’ ministries of health, agriculture, and defense to address potential biosecurity threats, such as by ensuring that countries keep inventories and management plans for dangerous pathogens stored in laboratories. Countries applied CDC’s expertise to ensure proper laboratory management and biosafety certification, which are imperative for ensuring the integrity of the national laboratory system. This work is critical for preventing national and international public health emergencies by preventing potential biosecurity threats.

Trained field epidemiologists, laboratory technicians, and emergency responders are crucial for detecting and responding to public health threats early and effectively, and EOCs with incident management systems are essential for response coordination (*27*). In July 2014, when the major EVD outbreak was worsening in West Africa, CDC-trained disease detectives performed contact tracing on 894 contacts of EVD case-patients in Lagos, Nigeria (*27*); only 11 deaths in Nigeria resulted from this EVD outbreak, although models estimated thousands of deaths would have occurred without timely investigation and emergency management (*19*). This example illustrates the potential impact of GHSA implementation. Training disease detectives and developing effective incident management can mean the difference between small outbreaks that are quickly and effectively controlled and larger outbreaks with substantial global health implications. CDC established new FETPs in 16 Phase I countries to rapidly train disease detectives. CDC worked with Phase I countries to establish EOCs and train emergency response staff. A component of the training involved activating the EOC for exercises or real public health emergencies. These activations incorporated a multisectoral approach to bring together public health, animal health, border security, and other sectors. These efforts strengthen capacities and test countries’ abilities to respond to public health threats effectively and rapidly.

The accomplishments we describe have enhanced global health security, but GHSA relies on strong partnerships to sustain capacity-building efforts. CDC’s work has strengthened collaborations among countries, US government agencies, and international governments and organizations. While emphasizing a multisectoral approach for building GHSA capabilities, CDC uniquely provides direct technical assistance to MOHs, developing their expertise so they can sustain GHSA accomplishments. CDC worked with multiple partners, including national ministries of health, agriculture, and defense, to establish mechanisms for cross-sectoral communication and collaboration that are essential for outbreak prevention, detection, and response but did not exist before GHSA. CDC’s technical assistance complemented efforts by other nations and US government entities, including the US Agency for International Development, the Defense Threat Reduction Agency, and the US Department of Agriculture. Notably, the relatively small US investment in GHSA led to additional investments from other donor nations. For example, South Korea committed $100 million to build global health security capabilities in 13 countries (*28*).

In addition to technical assistance, CDC contributed to the development of the Joint External Evaluation (JEE) tool, an independent, transparent evaluation that employs 48 indicators to measure progress toward GHSA and IHR 2005 targets (*12*). A benefit of the JEE is its potential for standardizing metrics and streamlining CDC’s technical assistance across multiple countries. CDC worked with the World Health Organization and other partners to develop a library of achievements needed to advance from one level of capacity to higher levels (*29*). Most of the accomplishments we describe (Tables 1–4) are among the milestones in the library, with related JEE indicators associated with each. This work demonstrates the feasibility and effectiveness of these activities in the field. The milestones library, together with JEE scores, helps CDC standardize and streamline technical assistance to complement activities planned by other partners. Although the administrative efforts required to undergo the JEE delayed CDC’s activities in some countries, the JEE process has now been operationalized, and countries have built their evaluation capacities by completing these baseline assessments. As of September 2017, a total of 58 countries, including 14 Phase I countries, completed the JEE with CDC support, identifying countries’ IHR 2005 capabilities and the explicit gaps in need of prioritization.

Our report has a few limitations. First, this report is not comprehensive of all CDC’s GHSA achievements. It focuses on CDC-supported country accomplishments in 17 countries, excluding CDC’s GHSA achievements beyond Phase I countries, including in Ebola preparedness countries where CDC prioritized GHSA work to build detection and preparedness capabilities to prevent cross-border spillover of EVD and other disease threats. Also, in initial analyses, evaluators determined that some accomplishments had been achieved by <6 Phase I countries and thus omitted these from the list provided to CDC field staff for validation; however, >6 countries might have achieved some of these by March 2017. Furthermore, CDC field staff validated accomplishments subjectively based on their interpretations of standardized language, potentially resulting in underreporting or overreporting. Despite these limitations, this report describes substantial accomplishments in 17 countries that resulted directly from the technical assistance provided by CDC. These achievements align with GHSA targets, suggesting that CDC has helped these countries move closer to attaining IHR 2005 core capacities, thus creating a safer world.

In conclusion, GHSA was launched with a goal of making the world safer from infectious disease threats by improving countries’ IHR 2005 core capacities (*4*). CDC’s efforts have been critical as part of a long-term process of building and sustaining global health security capacity in countries with less-developed public health systems. Initial accomplishments have laid the groundwork for further GHSA advancement in these 17 countries, and lessons learned might improve the efficiency of GHSA implementation in additional countries. Ongoing GHSA implementation offers an alternative to the cycle of panic and neglect that describes the current response to pandemic threats (*30*). The initial successes we describe demonstrate that strategic appropriation of technical and financial resources can accelerate progress toward GHSA targets and global achievement of IHR 2005 core capacities. CDC’s continuing work with partner countries ensures sustainability and further progress rather than regression. Furthermore, investments in global health security have been shown to have positive health, security, and economic impacts (*31*,*32*). These improvements in international capacity to rapidly detect, respond to, and control infectious disease outbreaks and other public health threats at their sources translate into enhanced global health security, because fewer public health threats can spread throughout a country and reach other nations, including the United States.

## References

[1] World Health Organization. Report of the Review Committee on the Role of the International Health Regulations (2005) in the Ebola Outbreak and Response [cited 2017 Aug 2]. http://www.who.int/ihr/review-committee-2016

[2] World Health Organization. Report of the Review Committee on the Functioning of the International Health Regulations (2005) in Relation to Pandemic (H1N1) 2009 [cited 2017 Aug 2]. http://www.who.int/ihr/publications/RC_report

[3] Jonas O. Pandemic risk. Background paper for the World Development Report [cited 2017 Aug 2]. http://siteresources.worldbank.org/EXTNWDR2013/Resources/8258024-1352909193861/8936935-1356011448215/8986901-1380568255405/WDR14_bp_Pandemic_Risk_Jonas.pdf

[4] Katz R, Sorrell EM, Kornblet SA, Fischer JE. Global Health Security Agenda and the International Health Regulations: moving forward. Biosecur Bioterror. 2014;12:231–8. 10.1089/bsp.2014.0038 25254911

[5] Global Health Security Agenda. [cited 2017 Apr 27]. https://www.GHSAgenda.org

[6] Centers for Disease Control and Prevention. US Commitment to the Global Health Security Agenda [cited 2017 Apr 27]. https://www.cdc.gov/globalhealth/security/pdf/ghs_us_commitment.pdf

[7] Schuchat A, Tappero J, Blandford J. Global health and the US Centers for Disease Control and Prevention. Lancet. 2014;384:98–101. 10.1016/S0140-6736(14)60570-5 24998008 PMC7137992

[8] Centers for Disease Control and Prevention. Global Health Security Agenda: action packages [cited 2017 Aug 2]. https://www.cdc.gov/globalhealth/security/actionpackages/default.htm

[9] World Health Organization. International Health Regulations (2005). 3rd ed. [cited 2017 Aug 2]. http://apps.who.int/iris/ bitstream/10665/246107/1/9789241580496-eng.pdf

[10] Borchert JN, Tappero JW, Downing R, Shoemaker T, Behumbiize P, Aceng J, et al.; Centers for Disease Control and Prevention (CDC). Rapidly building global health security capacity—Uganda demonstration project, 2013. MMWR Morb Mortal Wkly Rep. 2014;63:73–6. 24476978 PMC4584897

[11] Tran PD, Vu LN, Nguyen HT, Phan LT, Lowe W, McConnell MS, et al.; Centers for Disease Control and Prevention (CDC). Strengthening global health security capacity—Vietnam demonstration project, 2013. MMWR Morb Mortal Wkly Rep. 2014;63:77–80. 24476979 PMC4584898

[12] World Health Organization. Joint External Evaluation tool: International Health Regulations (2005) [cited 2017 Aug 2]. http://apps.who.int/iris/handle/10665/204368

[13] Balajee SA, Arthur R, Mounts AW. Global health security: building capacities for early event detection, epidemiologic workforce, and laboratory response. Health Secur. 2016;14:424–32. 10.1089/hs.2015.0062 27898218

[14] Centers for Disease Control and Prevention. Field Epidemiology Training Program: how we train [cited 2017 Apr 27]. https://www.cdc.gov/globalhealth/healthprotection/fetp/train.html

[15] Ameme DK, Nyarko KM, Kenu E, Afari EA. Strengthening surveillance and response to public health emergencies in the West African sub-region: the role of Ghana FELTP. Pan Afr Med J. 2016;25(Suppl 1). 10.11604/pamj.supp.2016.25.1.10579 PMC5257012 28149432

[16] Andre A, Lopez A, Perkins S, Lambert S, Chace L, Noudek N, et al. Frontline field epidemiology training programs as a strategy to improve disease surveillance and response. Emerg Infect Dis. 2017;23:166. 10.3201/eid2313.170803 29155657 PMC5711307

[17] Centers for Disease Control and Prevention. CDC Emergency Operations Center: Public Health Emergency Management Fellowship [cited 2017 Apr 27]. https://www.cdc.gov/phpr/eoc/emergencymanagementfellowship.htm

[18] American Veterinary Medical Association. One Health: a new professional imperative. One Health Initiative Task Force Final Report. Schaumburg (IL): The Association; 2008 [cited 2017 Aug 2]. https://www.avma.org/KB/Resources/Reports/Pages/One-Health.aspx

[19] Fasina FO, Shittu A, Lazarus D, Tomori O, Simonsen L, Viboud C, et al. Transmission dynamics and control of Ebola virus disease outbreak in Nigeria, July to September 2014. Euro Surveill. 2014;19:20920. 10.2807/1560-7917.ES2014.19.40.20920 25323076

[20] Kekulé AS. Learning from Ebola virus: how to prevent future epidemics. Viruses. 2015;7:3789–97. 10.3390/v7072797 26184283 PMC4517126

[21] Smolinski MS, Crawley AW, Olsen JM. Finding outbreaks faster. Health Secur. 2017;15:215–20. 10.1089/hs.2016.0069 28384035 PMC5404242

[22] Baize S, Pannetier D, Oestereich L, Rieger T, Koivogui L, Magassouba N, et al. Emergence of Zaire Ebola virus disease in Guinea. N Engl J Med. 2014;371:1418–25. 10.1056/NEJMoa1404505 24738640

[23] Maïnassara HB, Paireau J, Idi I, Pelat J-PM, Oukem-Boyer OOM, Fontanet A, et al. Response strategies against meningitis epidemics after elimination of serogroup A meningococci, Niger. Emerg Infect Dis. 2015;21:1322–9. 10.3201/eid2108.141361 26196461 PMC4517723

[24] Dowell SF, Blazes D, Desmond-Hellmann S. Four steps to precision public health. Nature. 2016;540:189–91. 10.1038/540189a

[25] Sealy TK, Erickson BR, Taboy CH, Ströher U, Towner JS, Andrews SE, et al. Laboratory Response to Ebola - West Africa and United States. MMWR Suppl. 2016;65:44–9. 10.15585/mmwr.su6503a7 27389781

[26] Olmsted SS, Moore M, Meili RC, Duber HC, Wasserman J, Sama P, et al. Strengthening laboratory systems in resource-limited settings. Am J Clin Pathol. 2010;134:374–80. 10.1309/AJCPDQOSB7QR5GLR 20716792

[27] Wolicki SB, Nuzzo JB, Blazes DL, Pitts DL, Iskander JK, Tappero JW. Public health surveillance: at the core of the Global Health Security Agenda. Health Secur. 2016;14:185–8. 10.1089/hs.2016.0002 27314658 PMC6937158

[28] Kim HS. Korea dedicates $100 million to help poor countries fight infectious disease [cited 2017 Aug 2]. http://www.koreatimesus.com/s-korea-dedicates-100-million-to-help-poor-countries-fight-infectious-diseases

[29] GHSA standardized milestone library [cited 2017 Oct 6]. https://www.ghsagenda.org/docs/default-source/default-document-library/GHSA-Milestone-Library.pdf

[30] The World Bank. Transcript: World Bank Group opening press conference by President Jim Yong Kim at the 2017 WBG/IMF Spring Meetings. Washington: World Bank; 2017 [cited 2017 Aug 2]. http://www.worldbank.org/en/news/speech/2017/04/20/2017-wbgimf-spring-meetings-world-bank-group-opening-press-conference-by-president-jim-yong-kim

[31] Gostin LO, Ayala AS. Global health security in an era of explosive pandemic potential. Journal of National Security Law and Policy. 2017;9:1.

[32] Sands P, El Turabi A, Saynisch PA, Dzau VJ. Assessment of economic vulnerability to infectious disease crises. Lancet. 2016;388:2443–8. 10.1016/S0140-6736(16)30594-3 27212427 PMC7159273

